# Comment on Murphy et al.: Pediatric orthopaedic lower extremity trauma and venous thromboembolism

**DOI:** 10.1007/s11832-016-0726-8

**Published:** 2016-03-26

**Authors:** Salih Marangoz

**Affiliations:** Department of Orthopaedics and Traumatology, Koc University School of Medicine, Koc University Hospital, Davutpaşa Cad. No.4, Topkapı, Istanbul, Turkey

Dear Sir or Madam,

I read with great interest the article by Murphy et al. [1] entitled “Pediatric orthopaedic lower extremity trauma and venous thromboembolism.” The authors performed extensive research on the incidence of a relatively rare complication seen in children. They conclude in their final paragraph that, according to their analysis, adolescents older than 12 years of age appear to experience a higher incidence of venous thromboembolism (VTE) than infants and children do. However, they do not give any data in support of this conclusion. Furthermore, in the “Results” section, they give the average age of the patients as 12.9 years with a range of 0–19 years. Was there VTE in an infant less than 1 year of age due to lower extremity trauma, or was that a mistake? Given this asymmetric distribution, I wonder what the median age was in their study population? The authors underline that adolescents and polytrauma patients with injuries of the femur/femoral neck, tibia/ankle, and pelvis were more commonly affected. It would be interesting to know more about the data that they did not include in the manuscript but which helped to lead them to these conclusions. Only 15 % of the VTE patients suffered polytrauma. How did the authors draw such a conclusion from the given data? Was a denominator available for the incidence of all polytrauma patients versus that of nonpolytrauma patients with lower extremity trauma? In polytrauma patients, additional organ injuries should be given as well as mentioning the number of diagnoses (in the range between 2 and 4). There is inconsistency in the manuscript regarding the number of patients who had at least one anticoagulant and were simultaneously coded with VTE and lower extremity orthopedic trauma. The number is given as 121 of 167 patients in the last paragraph of the “Results” section. However, if the number of patients who were treated with low-molecular-weight heparin, warfarin, or aspirin is added to the 24 patients who had more than one anticoagulant coded during the encounter, the total is 174 patients. In addition, all of the percentages of patients treated with individual anticoagulants were calculated using a denominator of 167, which should be 121 according to the first sentence of the last paragraph of the “Results” section.

The authors provide a comprehensive review of the previous publications in the “Discussion,” but some of the reference citations are erroneous. The second paragraph of the “Discussion” starts by focusing on Georgopoulos et al.’s article [2], which is the ninth reference in the reference list at the end of the paper, whereas it is cited in the text as the eighth reference in the list. Also, Georgopoulos et al. found 74 cases, not 71, corresponding to an incidence of 0.0515 %, not 0.015 % as Murphy et al. mention in their article. The first sentence on page 383 starts with a discussion of Greenwald et al.’s article [3], which is the sixth reference in the reference list but is cited in the text as the fourth reference in the list. The third paragraph of the “Discussion” also contains mismatched reference citations. An article by Sabharwal and coauthors [4] surveying members of the Pediatric Orthopaedic Society of North America is the seventh reference in the reference list at the end of the paper but is cited in the text as the fifth reference in the list. The follow-up survey by Sabharwal et al. [5] is given as the eighth reference in the list, but is cited as the sixth. Finally, the last sentence of the fourth paragraph of the “Discussion” reports a survey by Sabharwal et al. [4] on using low-molecular-weight heparin to treat VTE; this is the seventh reference in the list but is cited as the ninth. It would be better if these citation errors had been corrected and brought to the reader’s attention through a clarification.

To summarize, I think that Murphy et al. investigated a rare but important complication, but our understanding of the relevant pathophysiology would have been aided by providing more detailed clinical and demographic data along with the statistics included in the manuscript.
